# Supplementary material for: Computational Characterization of the Dish-In-A-Dish, A High Yield Culture Platform for Endothelial Shear Stress Studies on the Orbital Shaker
Source: Micromachines (Basel). 2020 May 29;11(6):552. doi: 10.3390/mi11060552 (PMC7345652; doi:10.3390/mi11060552)
Supplement: Supplementary file 1 [file micromachines-11-00552-s001.pdf]

micromachines

Supplemental information 1, mesh sensitivity analysis

In this study, to set up the computational fluid dynamics (CFD) model, first mesh sensitivity analysis was carried out on the largest structure (dish diameter = 134 mm), which is the extreme case. Four types of mesh size were chosen for mesh sensitivity analysis study (i.e. 1.0 mm, 1.5 mm, 2.0 mm and 2.5 mm). The CFD model was run in ANSYS CFX under the following conditions:

- orbital diameter = 10 mm
- orbital rotational speed = 200 RPM
- initial medium height = 3 mm
- time step = 0.01 sec and whole-time length = 3.0 sec
- incompressible and Newtonian fluid with a dynamic viscosity (μ) of 0.7 mPa·s
- convergence criteria of root-mean-square residual of the mass and momentum < 10^{-4}.

The figure below presented the average WSS, minimum WSS and maximum WSS under 4 types of mesh size at the time point of 2.1 sec, when the fluid flow was stable. If set the mesh size of 1.0 mm as reference, the mesh sizes of 1.5 mm, 2.0 mm and 2.5 mm have the relative difference of: (i) 6.6%, 19.3% and 26.0% respectively for average WSS; (iii) 6.3% 15.6% and 50.0% respectively for minimum WSS; (iii) 6.3%, 22.3% and 28.3% respectively for maximum WSS. Furthermore, the CFD models were run on a computer with 16 GB RAM and 8 cores center processing unit (CPU): Intel i7-8700 @ 3.2 GHz. The CPU time for running the model was recorded as 29 hours 50 minutes, 5 hours 15 minutes, 2 hours 40 minutes and 1 hours 18 minutes for mesh size of 1.0 mm, 1.5 mm, 2.0 mm and 2.5 mm, respectively. Therefore, considering the CPU time and WSS accuracy, mesh size of 1.5 mm was chosen for meshing all the structures in this study.

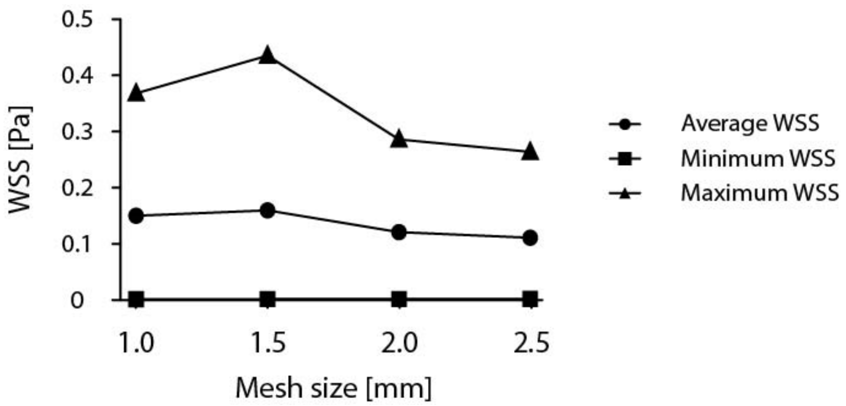

Supplemental information Figure 1. WSS results influenced by mesh sizes on the structure with the diameter of 134 mm.

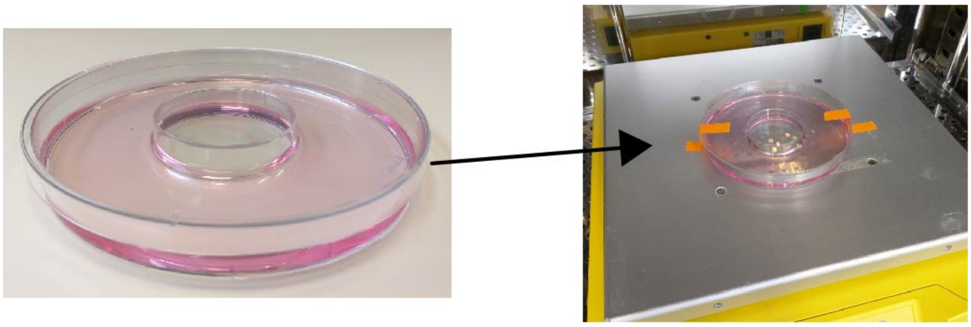

Figure S1. Experimental design. Fabricated DiaD and the experimental design. The DiaD is fixed on the orbital shaker do avoid drift.

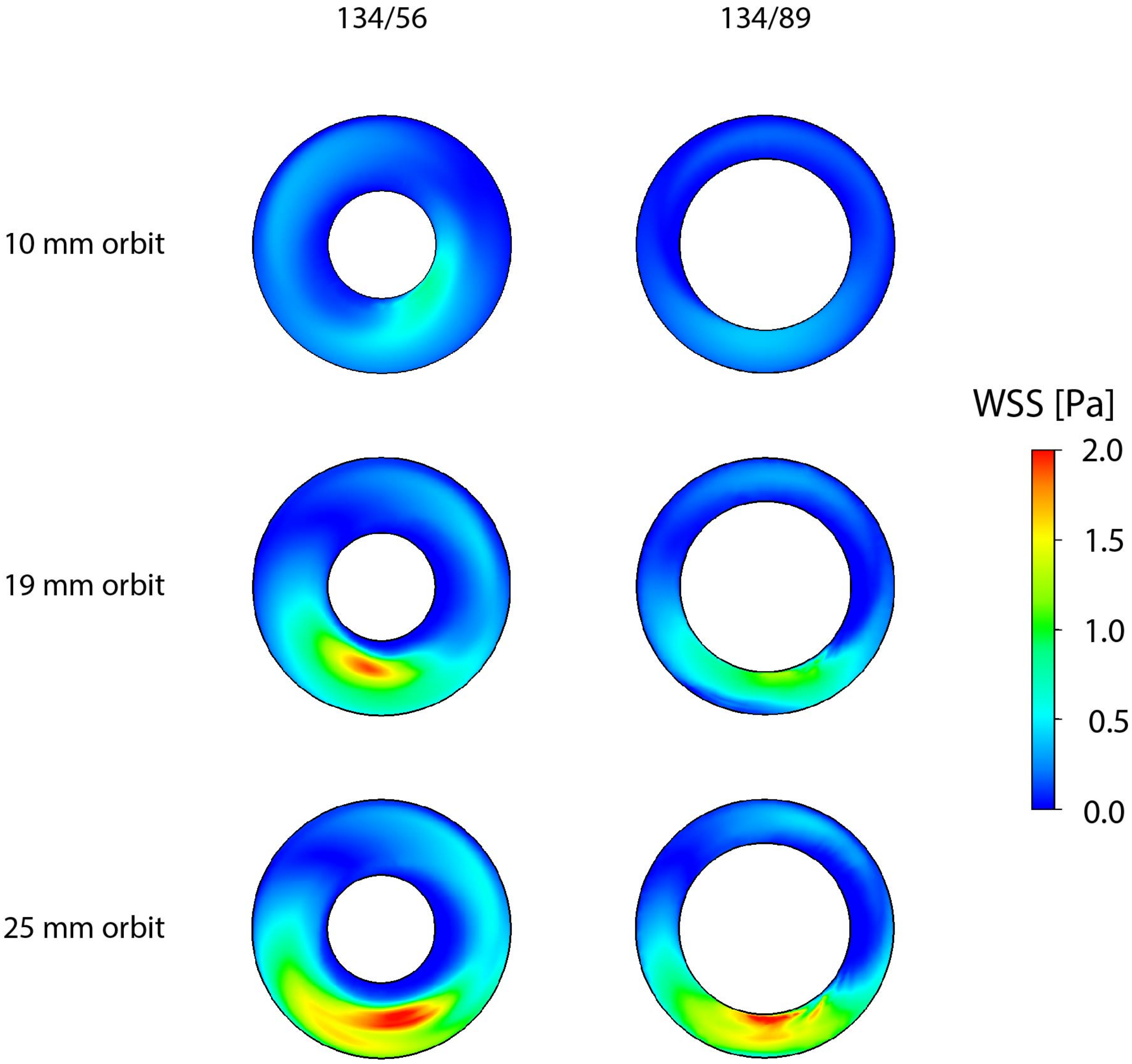

Figure S2. Shear distribution controlled by orbit size. Shear stress distribution heat maps in the 134/56 and 134/89 annular dish on orbital shakers with an orbit size of 10, 19 or 25 mm. The graphical representation scales from 0 to 2.0 Pa to cover shear stress distributions for both high and low rotational speeds.

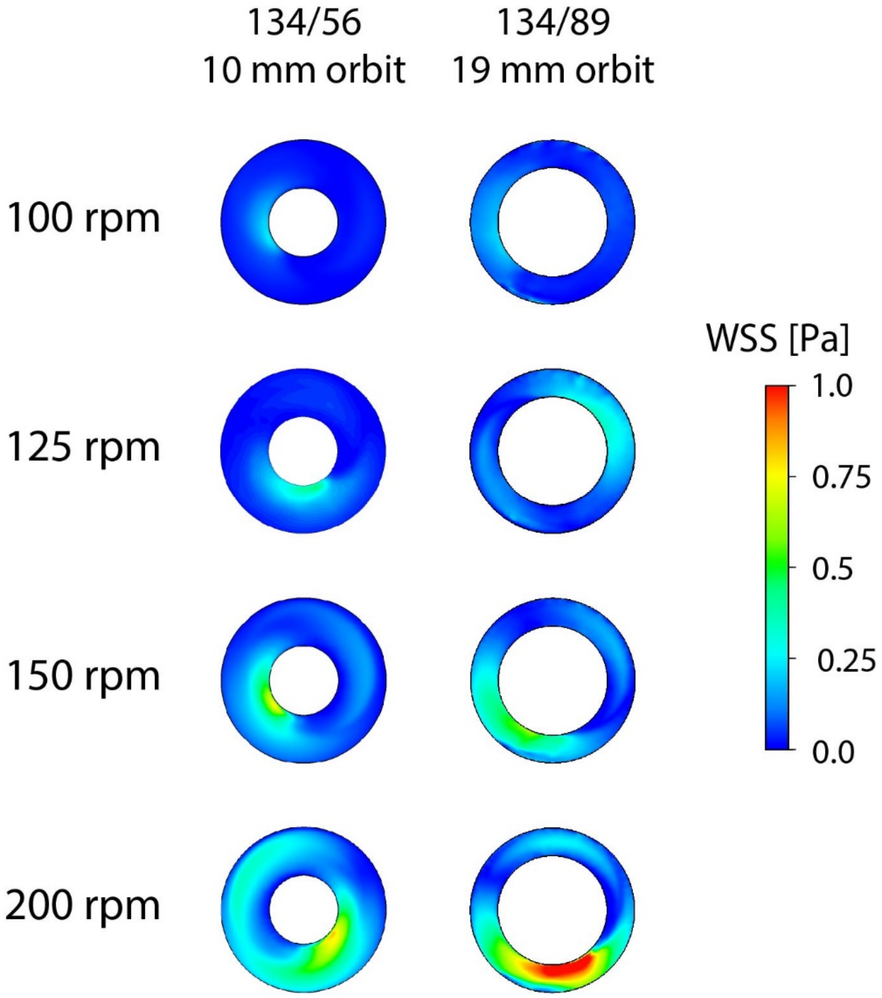

Figure S3. Shear distribution in the 134/56 and 134/89 annular dish in response to orbital speed. Heat maps of the shear stress distribution in the 134/56 (10 mm orbit) and 134/89 (19 mm orbit) annular dish on orbital shakers, swirled at an orbital speed of 100, 125, 150 and 200 rpm. The graphical representation scales from 0 to 1.0 Pa to cover shear stress distributions for both high and low rotational speeds.

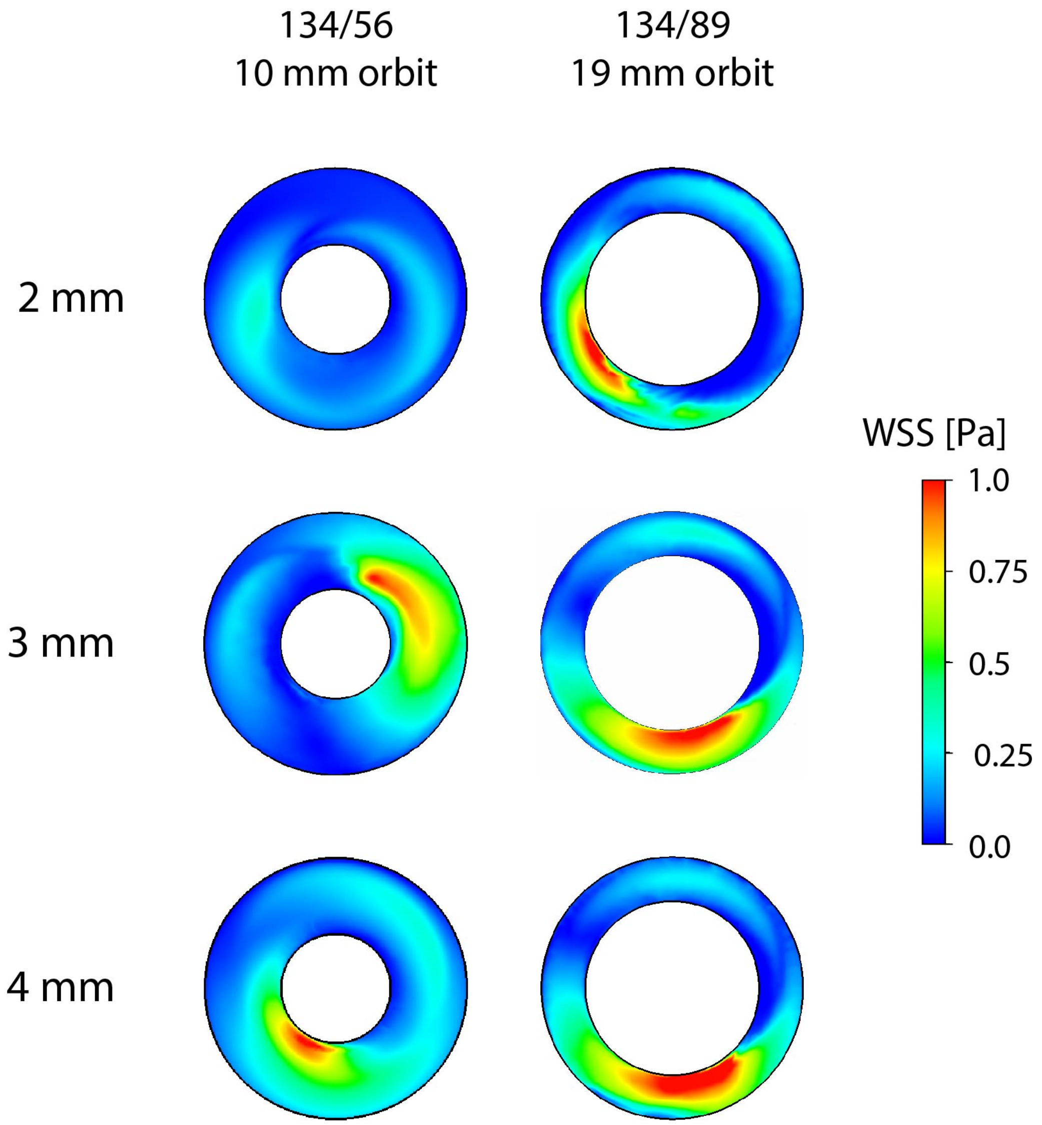

Figure S4. Shear distribution in the 134/56 and 134/89 annular dish in response to fluid height. Shear stress distribution heat maps in the 134/56 (10 mm orbit) and 134/89 (19 mm orbit) annular dish on the orbital shaker with a starting medium height of 2, 3 or 4 mm culture medium. The graphical representation scales from 0 to 1.0 Pa to cover shear stress distributions for both high and low rotational speeds.

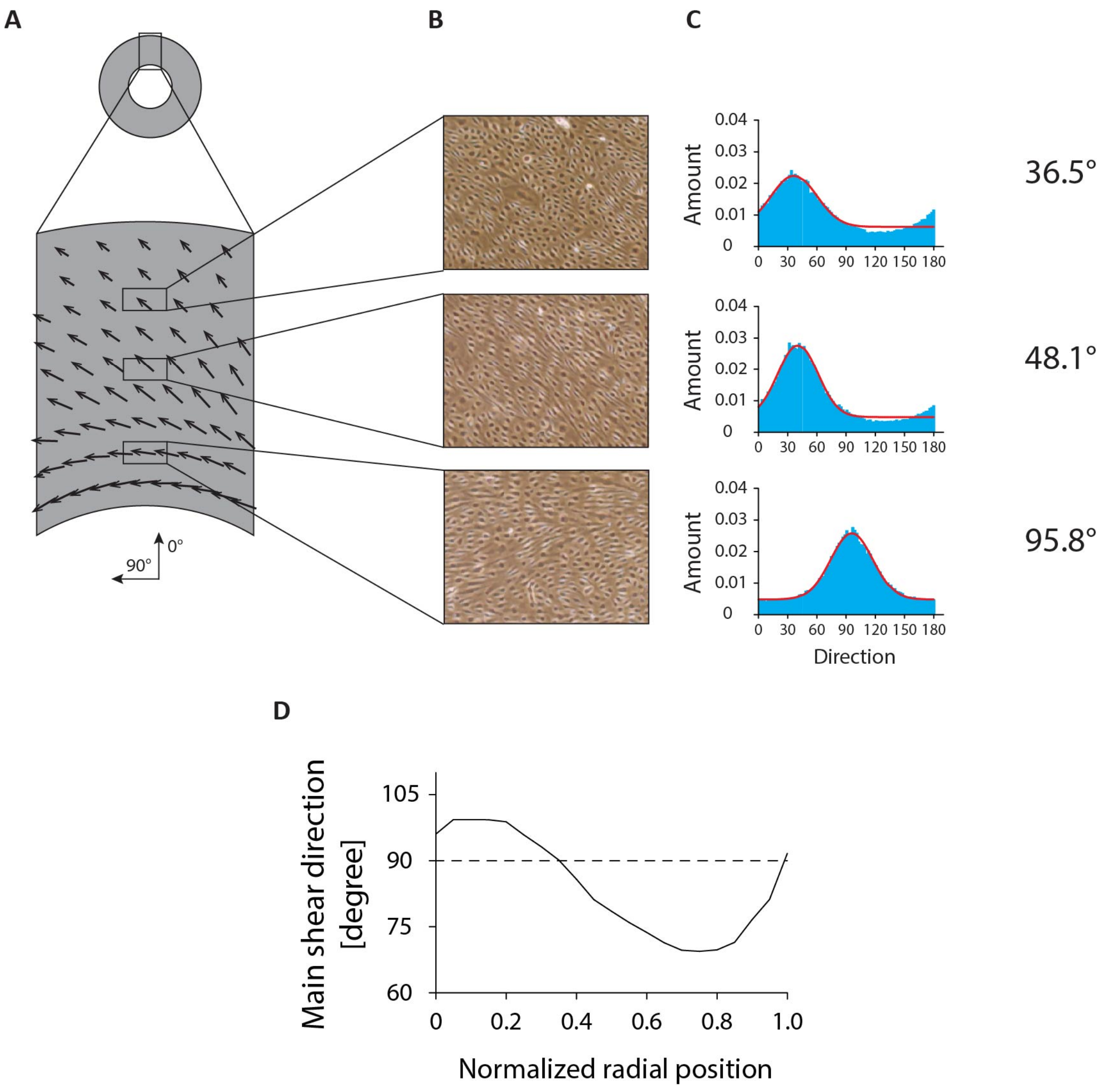

Figure S5. Endothelial alignment in the annular dish. (**A**) Graphical representation of the net shear stress vector in a 134/56 annular dish at 200 rpm, 10 mm orbit, and 3 mm fluid level. (**B**) Representative widefield images of ECs at different positions in a 134/56 annular dish after a 24-hour culture period under flow. The ECs align in the direction of the net flow direction. (**C**) Quantification of the cellular alignment. (**D**) The main shear stress direction is shown as a function of the radial position in the annular dish. The dashed line at 90 degrees indicates the orbital direction.

Table S1. Primers used in gene expression analysis.

Gene	Forward	Reverse
GAPDH	Reference gene assay (HK-SY-hu, Primer design, UK)	
eNOS	CAGCACAAGAGTTATAAGATCCGC	GCACTGTCTGTGTTACTGGACT
KLF2	CATCTGAAGGCGCATCTG	CGTGTGCTTTCGGTAGTGG
CHD2	AGGGGACCTTTTCCTCAAGA	CAATGTCAATGGGGTTCTCC
MCP1	CAGCCAGATGCAATCAATGCC	TGGAATCCTGAACCCACTTCT
